# Enhanced Efflux Pump Expression in *Candida* Mutants Results in Decreased Manogepix Susceptibility

**DOI:** 10.1128/AAC.00261-20

**Published:** 2020-04-21

**Authors:** Sean D. Liston, Luke Whitesell, Mili Kapoor, Karen Joy Shaw, Leah E. Cowen

**Affiliations:** a Department of Molecular Genetics, University of Toronto, Toronto, Ontario, Canada; b Amplyx Pharmaceuticals, San Diego, California, USA; c Hearts Consulting Group, San Diego, California, USA

**Keywords:** APX001, fosmanogepix, manogepix, APX001A, Gwt1, antifungal, GPI anchor, glycosylphosphatidylinositol, antifungal therapy, efflux

## Abstract

Manogepix is a broad-spectrum antifungal agent that inhibits glycosylphosphatidylinositol (GPI) anchor biosynthesis. Using whole-genome sequencing, we characterized two efflux-mediated mechanisms in the fungal pathogens Candida albicans and Candida parapsilosis that resulted in decreased manogepix susceptibility. In C. albicans, a gain-of-function mutation in the transcription factor gene *ZCF29* activated expression of ATP-binding cassette transporter genes *CDR11* and *SNQ2*. In C. parapsilosis, a mitochondrial deletion activated expression of the major facilitator superfamily transporter gene *MDR1*.

## Abstract (precis)

Manogepix is a broad-spectrum antifungal agent that inhibits glycosylphosphatidylinositol (GPI) anchor biosynthesis. Using whole-genome sequencing, we characterized two efflux-mediated mechanisms in the fungal pathogens Candida albicans and Candida parapsilosis that resulted in decreased manogepix susceptibility. In C. albicans, a gain-of-function mutation in the transcription factor gene *ZCF29* activated expression of ATP-binding cassette transporter genes *CDR11* and *SNQ2*.

## INTRODUCTION

Invasive fungal infections cause significant mortality and morbidity in humans, killing >1.5 million people annually (1, 2). A rapidly growing immunocompromised population is at particular risk, including those undergoing chemotherapy or solid organ transplantation or those infected with HIV (3–5). Current antifungal treatments are limited to three major classes of drugs: azoles, polyenes, and echinocandins (6). Issues of safety, tolerability, and the evolution of fungal drug resistance necessitate the development of antifungals with new mechanisms of action.

Fosmanogepix (formerly APX001, formerly E1211) is a novel intravenous (i.v.) and orally available *N*-phosphonooxymethyl prodrug that is currently in clinical development for the treatment of life-threatening invasive fungal infections that are often resistant to standard-of-care antifungal therapy (ClinicalTrials identifier NCT03604705) (7, 8). Fosmanogepix is converted by systemic phosphatases to the active moiety, manogepix (MGX; APX001A, formerly E1210) (9). MGX targets the essential fungal acyltransferase Gwt1 (10), blocking inositol acylation of glycosylphosphatidylinositol (GPI) anchors and trafficking of GPI-anchored proteins from the endoplasmic reticulum (ER) (11, 12). GPI anchors are attached to proteins in the ER and mediate their trafficking and attachment to the cell surface (13). MGX does not inhibit the mammalian Gwt1 homolog, PIGW (12). Gwt1 inhibition halts fungal growth, activates unfolded protein stress responses, and alters the composition of the fungal cell wall to expose immunostimulatory β-(1→3)-glucans (14). MGX has activity against the major fungal pathogens Candida albicans (15), Candida auris (16), Cryptococcus neoformans (17), and Aspergillus fumigatus (18), as well as less common pathogens, including *Fusarium* and *Scedosporium* (19).

To further explore the therapeutic potential of fosmanogepix, it is important to understand the potential for evolution of drug resistance. Spontaneous and serial passage experiments revealed that the *GWT1* missense mutations V162A (heterozygous) and V163A in C. albicans and Candida glabrata, respectively, demonstrated 16- and 32-fold increases in MGX MIC values (20). These mutations are hypothesized to impede drug binding to the target and differ from essential catalytic residues in Gwt1 (10, 21). Off-target mutations driving increases in MGX MIC values are largely unknown, although mutations in *EMP24* suppress toxicity of the Gwt1 inhibitor gepinacin (14). Emp24 facilitates quality control of GPI assembly by directing mature GPI-anchored proteins from the ER to the Golgi complex (22). Emp24 loss of function is predicted to release immature GPI-anchored proteins that accumulate during Gwt1 inhibition (23).

Spontaneous mutants of C. albicans (strain 5-3) and C. parapsilosis (strain 5-2) were identified that demonstrated decreased susceptibility to MGX and fluconazole (FLC) (20). The C. albicans mutant demonstrated 4-fold and 2-fold increases in the MICs of MGX and FLC, respectively, versus MICS of the isogenic wild-type strain, while the C. parapsilosis mutant demonstrated 8-fold and 4-fold increased MICs of MGX and FLC, respectively, versus MICs of the isogenic wild-type strain (20) (Table 1). *In vitro* susceptibility assays were performed as described in CLSI M27-A3, except that the dilution scheme consisted of 2-fold serial dilutions from 5 μM to 0.0049 μM (1.792 μg/ml to 0.00175 μg/ml). MIC values were determined at 50% growth inhibition relative to that of drug-free controls at 48 h (24).

**TABLE 1:** C. albicans 5-3 and C. parapsilosis 5-2 demonstrate elevated MICs to MGX and FLC, while C. albicans 5-3 and C. albicans *ZCF29/ZCF29*^W986L^ are resistant to beauvericin

Background	Strain	Manogepix		Fluconazole		Beauvericin	
		MIC (μg/ml)	Fold change vs WT	MIC (μg/ml)	Fold change vs WT	MIC (μg/ml)	Fold change vs WT
C. albicans ATCC 90028	WT	0.0035		0.125		3.125	
	5-3	0.014	4	0.5	4	100	32
C. parapsilosis ATCC 22019	WT	0.007		2		25	
	5-2	0.056	8	4	2	25	1
C. albicans LC191	WT	0.0035		0.125		3.125	
	*ZCF29/ZCF29*^W986L^	0.028	8	0.25	2	100	32

**FIG 1:**
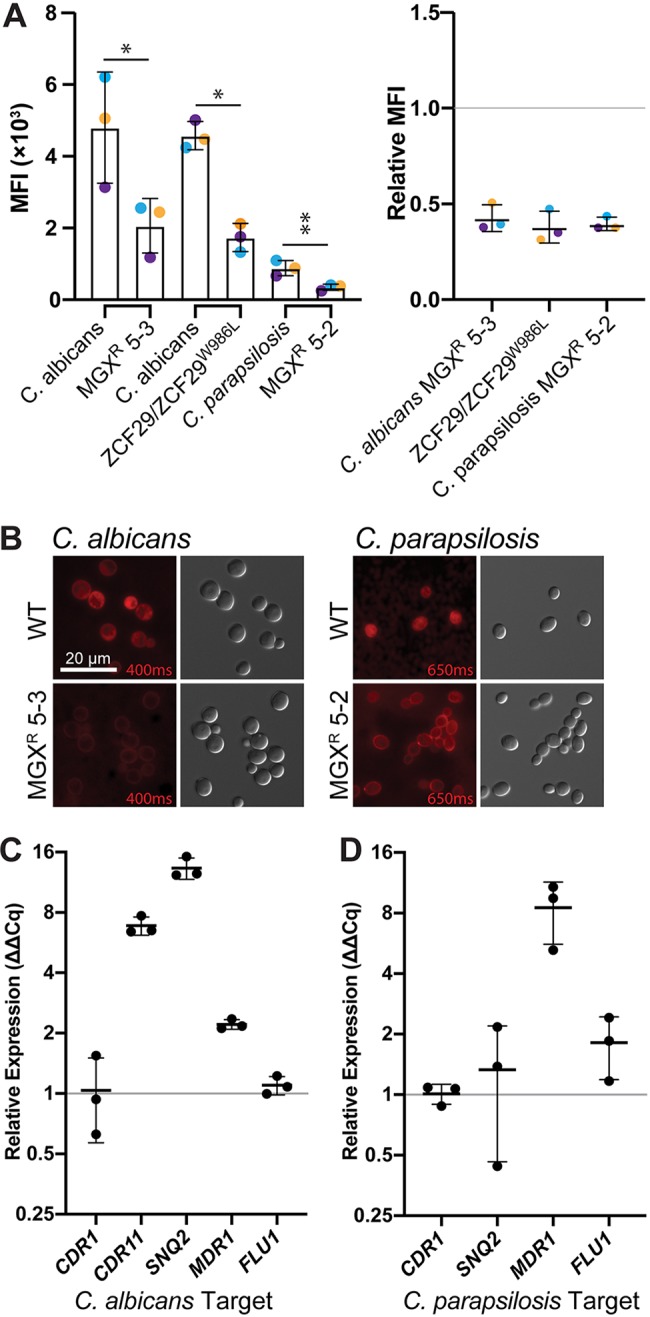
Drug efflux is activated in mutants of C. albicans and C. parapsilosis with reduced susceptibility to MGX. (A) C. albicans 5-3 and C. parapsilosis 5-2 mutants have reduced accumulation of the general efflux pump substrate Nile red. Nile red fluorescence was monitored by flow cytometry. (Left) Median fluorescence intensity (MFI; PE) ± standard deviation (SD) measured in 3 independent experiments (10,000 events/sample). (Right) Ratios of median fluorescence intensity for indicated mutant-wild type pair. Differences between groups were determined by ratio paired *t* test. *, *P *≤ 0.05; **, *P* ≤ 0.005. Colored points indicate experimental replicates. (B) Representative micrographs of C. albicans and C. parapsilosis wild-type strains and mutants with decreased MGX susceptibility stained with Nile red, prepared the same as for those in panel A. Exposure times (milliseconds) are indicated in red. (C) Relative transcript levels of *CDR11*, *SNQ2*, and *MDR1* but not *CDR1* or *FLU1* are upregulated in C. albicans MGX^r^ 5-3. RT-qPCR data are mean fold changes ± SDs from 3 biological replicates assayed in technical triplicates, normalized to *ACT1* and *GPD1*. (D) Transcript levels of *MDR1* but not *CDR1*, *SNQ2*, or *FLU1* are upregulated in C. parapsilosis MGX^r^ 5-2. Experiments were performed the same as for those in panel C and normalized to *ACT1*.

These two strains were not mutated in the *GWT1* gene; thus, we hypothesized that elevated MIC values for MGX and FLC could result from enhanced expression of multidrug efflux pumps, affecting the susceptibility to the structurally and mechanistically distinct antifungal agents. Although drug efflux has been frequently associated with antifungal resistance (25), it has not been described for MGX. To assess efflux in the strains with elevated MGX MIC values, we incubated cultures with Nile red, which accumulates in lipid membranes and is actively extruded from cells by efflux (26). Nile red is a substrate for the ATP-binding cassette (ABC) transporters Cdr1 and Cdr2, and the major facilitator superfamily transporter Mdr1 (26), which are the efflux pumps most frequently resulting in azole resistance in *Candida* (25). For these experiments, unit equivalents of an optical density at 600 nm (OD_600_) of 1 of log-phase cultures were washed 2 times in 1 ml buffer A (20 mM Na-HEPES, 150 mM NaCl, pH 7.5), resuspended in 1 ml buffer A, and incubated at 30°C for 2 h. Nile red was added to 7 μM and incubated for 1 h. Stained cells were washed 2 times in buffer A, and then efflux was initiated by addition of glucose to 1% (wt/vol). Nile red fluorescence was determined by flow cytometry after 30 min (Beckman CytoFLEX, phycoerythrin (PE) filter A01-1-0052; analysis with CytExpert 2.3) and visualized by fluorescence microscopy on a Zeiss AxioObserver.Z1 (Chroma Tech ET Cy5 filter). Both C. albicans and C. parapsilosis MGX mutants with elevated MIC values accumulated ∼60% less Nile red than their parental strains (Fig. 1A and B), implicating drug efflux in the decreased susceptibility to MGX.

To identify mutations responsible for activation of drug efflux, we sequenced the genomes of the MGX mutants using the Illumina MiSeq platform (Genewiz). Adaptor sequences and low-quality reads were removed using Trimmomatic v0.39 (27). Paired reads were assembled to *Candida* Genome Database (28) C_albicans_SC5314_A21 (29) and C_parapsilosis_CDC317 (30) by using Bowtie2 v2.3.5.1 (31) (Table 2). Missense single nucleotide variants (SNVs) between parental and mutant assemblies were detected using Mutect v1.1.7 (32) and SnpEff v2.6.3 (33) and validated by Sanger sequencing. Loss of heterozygosity or aneuploidy was not detected by the Yeast Mapping Analysis Pipeline (34) and CNV-seq (35).

**TABLE 2:** Fungal strains used in this study

Strain	Genotype	Reference or source
C. albicans		
ATCC 90028	Wild type	ATCC
ATCC 90028 5-3	*ZCF29*^W986L^/*ZCF29*	20
CaLC191 (DAY185)	*URA3/ura3*::*imm434 HIS1/his1*::*hisG ARG4/arg4*::*hisG*	45
CaLC3815	*URA3/ura3*::*imm434 HIS1/his1*::*hisG ARG4/arg4*::*hisG ZCF29*^W986L^/*ZCF29*	36
C. parapsilosis		
ATCC 22019	Wild type	ATCC
ATCC 22019 5-2	*capafmp06*Δ3780-5672	20

In C. albicans MGX^r^ 5-3, a single heterozygous SNV resulted in a W986L substitution in the Zn(II)_2_Cys_6_ transcription factor Zcf29. This gain-of-function mutation was previously identified in beauvericin-resistant C. albicans (36). Consistent with this connection, C. albicans MGX^r^ 5-3 demonstrated a 32-fold increase in the MIC value versus that in the wild-type (WT) strain to beauvericin, and an engineered *ZCF29*/*ZCF29*^W986L^ C. albicans mutant demonstrated an 8-fold increase in the MGX MIC value versus that in the WT strain (Table 1). Transcript levels for efflux pump genes were assessed using reverse transcriptase quantitative real-time PCR (RT-qPCR). RNA was extracted from 10 ml log-phase yeast extract-peptone-dextrose (YPD) cultures grown at 30°C by using a Qiagen RNeasy minikit. RNA was treated with Qiagen RNase-free DNase and reverse transcribed using Bio-Rad iScript cDNA synthesis kit. qPCR was performed using Thermo Fisher Scientific SYBR green master mix and oligonucleotide primers described in Table S2 in the supplemental material. Data were analyzed using Bio-Rad CFX Manager 3.1. Transcript levels of efflux genes *CDR11*, *SNQ2*, and *MDR1* were increased in MGX^r^ 5-3 by 6.8, 13.3, and 2.2-fold, respectively (Fig. 1C), consistent with transcriptional profiling of C. albicans *ZCF29/ZCF29*^W986L^ (36). Transcript levels of *CDR1* and *FLU1* were unchanged (Fig. 1C).

**FIG 2:**
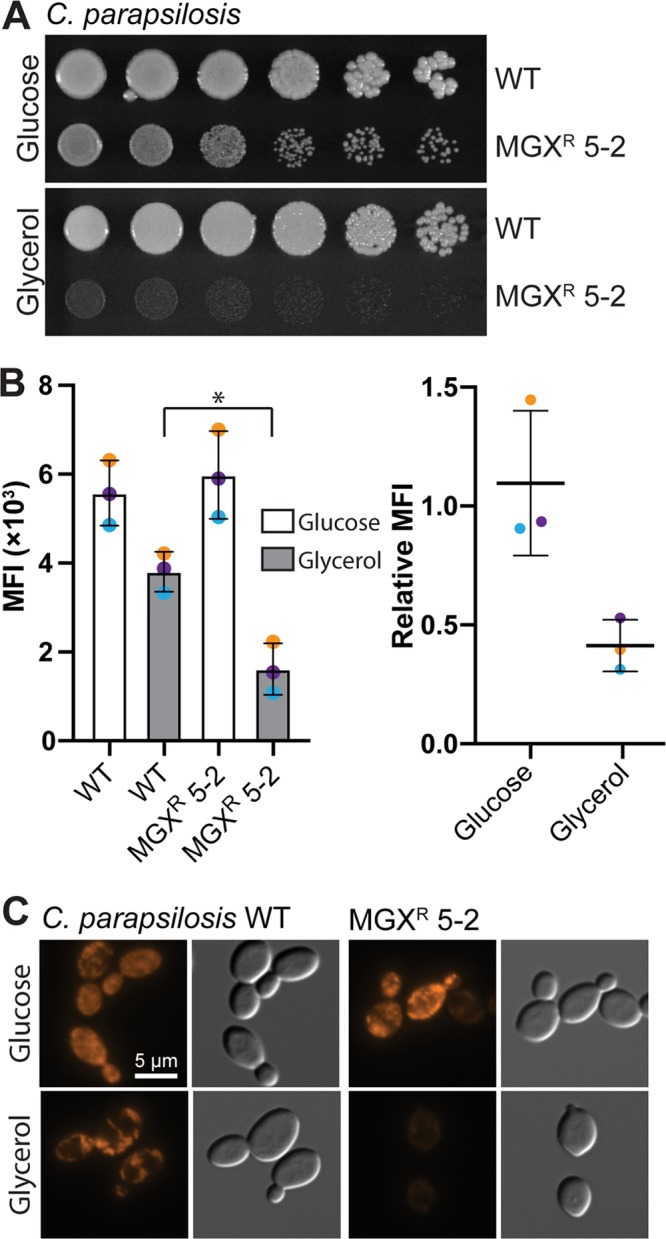
C. parapsilosis MGX^r^ 5-2 has a defect in mitochondrial function. (A) C. parapsilosis MGX^r^ 5-2 forms petite colonies on YPD agar and does not grow on YP-glycerol agar; 10-fold dilutions of stationary-phase cultures of C. parapsilosis were spotted on YP agar containing 2% (wt/vol) d-glucose or glycerol and then photographed after 48 h of growth at 30°C. (B) C. parapsilosis MGX^r^ 5-2 has reduced mitochondrial membrane potential when subcultured in medium containing 2% (wt/vol) glycerol. MitoTracker Red CMXRos fluorescence was monitored by flow cytometry. Data are median fluorescence intensities (DsRed) ± SDs from 3 independent experiments (10,000 events/sample). Differences between groups were determined by ratio paired *t* test. *, *P* ≤ 0.05. (C) Representative micrographs of MitoTracker Red-stained cells prepared the same as for those in panel B.

No missense SNVs were detected in C. parapsilosis MGX^r^ 5-2; however, a deletion was detected in the mitochondrial chromosome from bases 15430 to 17322. The boundaries of this deletion were defined using MitoDel v3.0 (37), which identified 2,087 reads across this junction. This deletion interrupts *CAPAFMP06* (*COX1*), which encodes cytochrome c oxidase, and *CAPAFMP06.3*/*CAPAFMP06.4*, intronic open reading frames (ORFs) to *COX1* that encode enzymes with predicted roles in mRNA splicing (38). Consistent with a respiratory defect, C. parapsilosis MGX^r^ 5-2 formed petite colonies on YP glucose-containing medium and did not grow on YP medium containing the nonfermentable carbon source glycerol (Fig. 2A). Furthermore, when subcultured in glycerol-supplemented synthetic complete medium for 2 h and then stained with 10 nM mitochondrial membrane potential-dependent stain MitoTracker Red CMXRos (Invitrogen) for 30 min, C. parapsilosis MGX^r^ 5-2 showed ∼60% reduced staining relative to that of the parental strain. MitoTracker Red fluorescence was quantified by flow cytometry (Fig. 2B) (Beckman CytoFLEX, DsRed filter A01-1-0053) and visualized by fluorescence microscopy (Fig. 2C) on a Zeiss AxioObserver.Z1 (Chroma Tech ET HQ DsRed filter).

Respiratory competence is linked to drug susceptibility in diverse fungal pathogens and is often driven by efflux pump overexpression (39). Indeed, transcript levels of *MDR1* were 8.5-fold upregulated in C. parapsilosis MGX^r^ 5-2 (Fig. 1C), consistent with a previously described petite mutant of C. albicans with decreased susceptibility to FLC (40, 41). In some Saccharomyces cerevisiae and C. glabrata petite mutants, the Pdr1/Pdr3 transcription factors induce expression of ABC transporter genes *PDR5* (ortholog of *CDR1*), *SNQ2*, and *YOR1* (42–44). This is not the case in C. parapsilosis MGX^r^ 5-2, as transcript levels of *CDR1* and *SNQ2* were unchanged (Fig. 1C).

In conclusion, we have identified two efflux-mediated mechanisms conferring reduced susceptibility to MGX in two *Candida* species. In C. albicans, a gain-of-function mutation in the transcription factor gene *ZCF29* activated expression of ABC transporter genes *CDR11* and *SNQ2*. In C. parapsilosis, a mitochondrial deletion activated expression of the major facilitator superfamily (MFS) transporter gene *MDR1*. The MIC of MGX was at maximum 0.0056 μg/ml, suggesting that these individual mutations may not result in clinically significant resistance. Additionally, loss of mitochondrial function is expected to impair virulence, as observed with some C. albicans petite mutants (41).

## Supplementary Material

Supplemental file 1
