# Supplementary material for: Enhanced Efflux Pump Expression in Candida Mutants Results in Decreased Manogepix Susceptibility
Source: Antimicrob Agents Chemother. 2020 Apr 21;64(5):e00261-20. doi: 10.1128/AAC.00261-20 (PMC7179633; doi:10.1128/AAC.00261-20)
Supplement: Supplemental file 1 [file AAC.00261-20-s0001.pdf]

Supplementary Data

Table S1. Summary statistics for genome assemblies.

**Assembly**	**Coverage (fold)**	**Reads**	**% bases with >15 coverage**
C. albicans ATCC90028	56.76	812808390	98.0
C. albicans ATCC90028 5-3	57.74	826918792	99.2
C. parapsilosis ATCC22019	147.79	1925694538	99.7
C. parapsilosis ATCC22019 5-2	144.17	1878546214	99.7

Table S2. Oligonucleotide primers used in this study.

Primer	Feature	Sequence (5′→3′)
oLC752	*C. albicans GPD1* Fw	agtatgtggagctttactggga
oLC753	*C. albicans GPD1* Rv	cagaaacaccagcaacatcttc
oLC2285	*C. albicans ACT1* Fw	gaccttgagatacccaattg
oLC2286	*C. albicans ACT1* Rv	cagcttgaatggaaacgtag
oLC8344	*C. parapsilosis COX1* Fw	actactttaaacatgcgtagtataggag
oLC8345	*C. parapsilosis COX1*Rv	gcaccatcaccttcaataaatcc
oLC8346	*C. parapsilosis COX1* seq	atcattacctgtattaacagctggtgttac
oLC8347	*C. albicans ZCF29* Fw	cctcaatctcaatatcgatcccaaag
oLC8348	*C. albicans ZCF29* Rv	tcattacttccactagctaactctgatc
oLC8349	*C. albicans ZCF29* seq	ggagctaaccctcagtattcatatg

oLC8379	*C. albicans CDR1* Fw	gtgctggttgttccacattg
oLC8380	*C. albicans CDR1* Rv	acatcaccacggtaatgacg
oLC8381	*C. albicans CDR11* Fw	aaagctattggaaacgctcg
oLC8382	*C. albicans CDR11* Rv	tttgactgtttggccacatg
oLC8383	*C. albicans SNQ2* Fw	acaaccttccaccgatgatg
oLC8384	*C. albicans SNQ2* Rv	cttctttattagtcacttgacggg
oLC8385	*C. parapsilosis CDR1* Fw	cacatgtcacaagtacctgg
oLC8386	*C. parapsilosis CDR1* Rv	caaatttcggtatccaacaccc
oLC8387	*C. parapsilosis SNQ2* Fw	cagttacccttattggtgctgg
oLC8388	*C. parapsilosis SNQ2* Rv	cagagatttctgccaatccc
oLC8389	*C. parapsilosis ACT1* Fw	aagattttgtccgaacgtgg
oLC8390	*C. parapsilosis ACT1* Rv	gaccatctggcaattcgtatg
oLC8391	*C. parapsilosis GPD1* Fw	ccattgcttacacattacccc
oLC8392	*C. parapsilosis GPD1* Rv	cccacagctaaagcaacaatg
oLC8465	*C. albicans MDR1* Fw	gcccattggttttcagtccg
oLC8466	*C. albicans MDR1* Rv	tggccaaacaaggactagca
oLC8467	*C. albicans FLU1* Fw	tggatagtccccgtcattgg
oLC8468	*C. albicans FLU1* Rv	aaagtgggaaaacagcccca
oLC8469	*C. parapsilosis MDR1* Fw	agtatacatggcatcggcgg
oLC8470	*C. parapsilosis MDR1* Rv	tctacccactgcggcatttt
oLC8471	*C. parapsilosis FLU1* Fw	ttggcttggttggagtggag
oLC8472	*C. parapsilosis FLU1* Rv	taccagccaatgcagaagca

6
